# Progression-free survival estimation of docetaxel-based second-line treatment for advanced non-small cell lung cancer: a pooled analysis from 18 randomized control trials

**DOI:** 10.3389/fonc.2024.1298786

**Published:** 2024-05-14

**Authors:** Chaithra N, Anisha Jain, Sahana C, Bhargav Shreevatsa, Saravanan Rajendrasozhan, Chandan Dharmashekar, Kuralayanapalya Puttahonnappa Suresh, Sharanagouda S. Patil, Pranav Singh, Prashant Vishwanath, Chandrashekar Srinivasa, Shiva Prasad Kollur, Chandan Shivamallu

**Affiliations:** ^1^ Division of Medical Statistics, Life Sciences and Natural Sciences Departments, JSS Academy of Higher Education and Research, Mysuru, Karnataka, India; ^2^ Department of Microbiology, JSS Academy of Higher Education and Research, Mysuru, Karnataka, India; ^3^ Department of Biotechnology and Bioinformatics, JSS Academy of Higher Education and Research, Mysuru, Karnataka, India; ^4^ Pathology, Microbiology and Immunology Department, School of Medicine, University of South Carolina, Columbia, SC, United States; ^5^ Department of Chemistry, Faculty of Science, University of Hail, Hail, Saudi Arabia; ^6^ Department of Spatial Epidemiology, ICAR-National Institute of Veterinary Epidemiology and Disease Informatics, Bengaluru, Karnataka, India; ^7^ ICAR-National Institute of Veterinary Epidemiology and Disease Informatics, Bengaluru, Karnataka, India; ^8^ Department of Medicine, Kasturba Medical College, Manipal Academy of Higher Education, Udupi, Karnataka, India; ^9^ Center of Excellence in Molecular Biology and Regenerative Medicine, Department of Biochemistry, JSS Medical College, JSS Academy of Higher Education and Research, Mysore, India; ^10^ Department of Studies in Biotechnology, Davangere University, Davangere, Karnataka, India; ^11^ School of Physical Sciences, Amrita Vishwa Vidyapeetham, Mysuru, Karnataka, India

**Keywords:** non-small cell lung carcinoma, lung neoplasms, progression-free survival, randomized control trials, meta analysis

## Abstract

**Background:**

Lung cancer is the foremost cause of cancer-related death globally, with non-small cell lung cancer (NSCLC) accounting for 85–90% of cases. Targeted therapy is the most essential therapeutic option for NSCLC, other common treatments include radiation therapy, surgery, chemotherapy, and immunotherapy.

**Objective:**

Our study objective was to estimate whether progression-free survival (PFS) is an outcome of NSCLC extracted from 18 randomized control trials (RCTs) with docetaxel as experimental group and antineoplastic agent, kinase inhibitor, and monoclonal antibodies as a control group.

**Methods:**

We selected relevant studies published between 2011 and 2022 using Google Scholar, PubMed, Scopus, Science Direct, and Cochrane Library. Advanced NSCLC, chemotherapy, RCT, docetaxel, and second-line treatment were the terms included in the search. A total of 9738 patients were evaluated from the 18 identified studies. We used the meta package of R Studio to perform the meta-analysis. Graphical funnel plots were used to evaluate publication bias visually.

**Results:**

Patients who underwent docetaxel-based therapy had a considerably longer PFS than those who got antineoplastic agents, kinase inhibitors, or monoclonal antibodies-based treatment. Patients in the standard treatment arm had a slightly longer PFS than those in the experimental therapy arm in the overall meta-analysis.

**Conclusion:**

Docetaxel outperformed monoclonal antibodies, antineoplastic agents, and kinase inhibitors in the second-line therapy of advanced NSCLC since PFS was extensively utilized.

## 1 Introduction

Cancer results from a complex multistep system including the accumulation of several gene mutations, which comprises encoding microRNA (1). Heredity ionizing radiation, chemical substances, alcohol, nitrates, estrogens, viruses, stress, and age are the main risk factors (2). Carcinoma, sarcoma, leukemia, lymphoma, and myeloma are types of cancer (3). According to the World Health Organization (WHO), it is the first or second largest cause of mortality before the age of 70 in 112 (of 183) nations, ranks third or fourth in another 23 countries, and was a major impediment to improving life expectancy in every country on the planet in 2019 (4).It has an impact on the high incidence of stroke and coronary heart disease mortality in many nations (5). HPV, HBV, HIV, and bacteria like Helicobacter pylori (stomach cancers) are infectious agents increasing the risk of cancer (6). The number of cancer cases is expected to increase from 979 786 in 2010 to 1 148 757 cases in 2020 (7). Lung cancer is the most recurrently diagnosed and the leading cause of cancer mortality. The two most common types of lung cancer are NSCLC and small cell lung cancer (SCLC). NSCLC makes for 80 to 85% of lung cancer cases, with SCLC accounting for the rest. Patients with lung cancer may be eligible for various therapies, including surgery, radiation, chemotherapy, and targeted therapy, depending on their stage. Targeted therapy is the most essential therapeutic option for NSCLC, other common treatments include radiation therapy, surgery, chemotherapy, and immunotherapy. Targeted therapies include monoclonal antibodies and small-molecule inhibitors. Specific mutations have been detected thanks to advances in genetics and biomarker testing, allowing doctors to better target treatment for individual patients (8, 9). Cigarette smoking is considered a significant hazard factor with an 82% mortality rate in males compared to females (10). It is asymptomatic in its early-stage, and patients diagnosed at an advanced stage experience a poor prognosis (11). The objective of our study was to estimate whether the PFS is an outcome of NSCLC, using data from 18 RCTs (12). PFS, the time from therapeutic initiation to disease progression, may be used as a measure of clinical benefit for drug approvals, depending on the condition and response observed (13).

## 2 Methods

We selected relevant studies published between 2011 and 2022 using Google Scholar, PubMed, Scopus, Science Direct, and Cochrane Library. Advanced NSCLC, chemotherapy, RCT, docetaxel, and second-line treatment were the terms included in the search.

Randomized trials including patients diagnosed with NSCLC that evaluated docetaxel with a kinase inhibitor, antineoplastic agents, and monoclonal antibodies for NSCLC were included. Docetaxel compared with other therapeutic agents except for kinase inhibitors, monoclonal antibodies and antineoplastic agents was considered exclusion criterion. Similarly, studies that compared docetaxel to other drugs were excluded, as well as early studies published as a series of articles by the same author with overlapping data, editorials, case reports, conference articles, experimental studies, and related studies that failed to provide significant findings. Authorship, publication bias, clinical trials, demographic attributes, histology characteristics, smoking status, treatment for each group, and adverse events were all extracted using a fixed standardized procedure. The conventional treatment in this trial was docetaxel, while the experimental arm was a kinase inhibitor, antineoplastic drug, or monoclonal antibody.

A comprehensive search approach was devised to reduce the risk of publishing bias. Graphical funnel plots were used to visually evaluate publication bias and the quality of RCTs.

Pooled HR was calculated with 95% CI. We used forest plots and inconsistency statistic [I2] to determine heterogeneity. The OR was the summary measure used for pooling the studies. Hedge’s method evaluates the effect size calculated by standard mean difference (SMD) given as Hedge’s g-value. The meta-analysis was summarized graphically using a forest plot. Meta package of R Studio (v2021.09.0) was used to perform the meta-analysis.

## 3 Results

**Figure 1:**
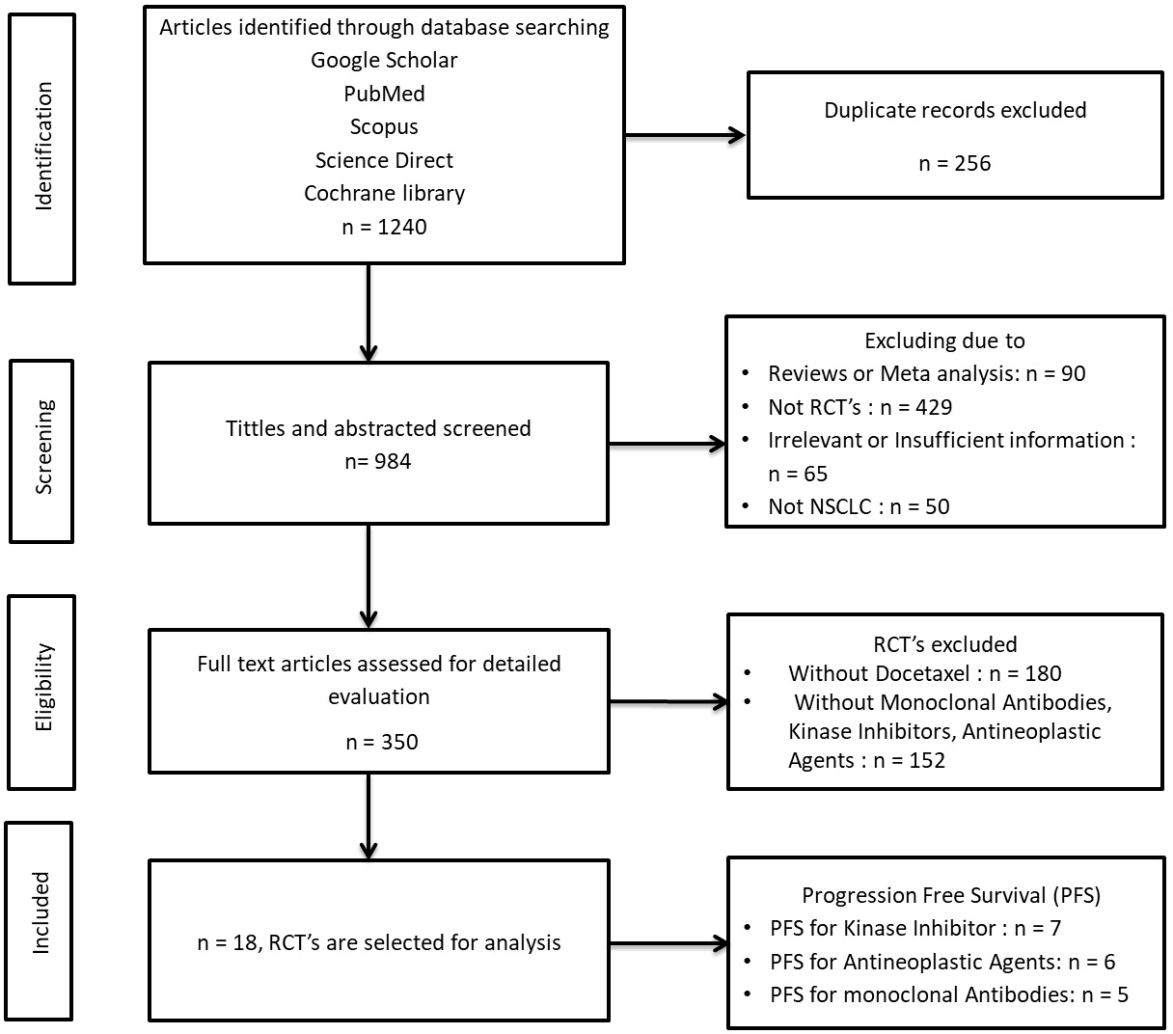
Flowchart of the study selection steps.

The details of study selection criteria followed for the meta-analysis of drug intervention prevalence are given in **Figure 1**. The number of published articles was 1240, of which 256 were rejected for duplication in one or the other form, 68 were excluded since reviews or meta-analysis and 429 were excluded as non-randomized control trials, 65 were excluded due to Irrelevant or Insufficient information and 50 excluded because of not NSCLC. Then after filtering 350 randomized control trials were selected for detailed evaluation, in which 180 were excluded which were treatment arms without docetaxel, and 152 were excluded which were without monoclonal antibodies, kinase inhibitors, and antineoplastic agents. Hence, finally 18 Randomized control trials were selected for the study.

The characteristics of selected RCTs of meta-analysis are given in **Table 1**. Six RCTs phase 3 data for the antineoplastic agent classes of intervention were analyzed, with 850 the maximum number of patients recorded with a median age of 64 and PFS as the primary endpoint. Data from seven phase 2 and 3 RCTs were analyzed for the kinase inhibitor class of intervention, with 1314 the highest number of patients having a primary endpoint of PFS with a median age of 60. The remaining five RCTs were phase 2 and 3 monoclonal antibody class intervention data analyzed with PFS as the main endpoint. The highest number of patients recorded was 1253 with a median age of 61.5 years.

**Table 1:** Characteristics of the selected RCTs for meta-analysis.

Sl. no	Study reference	Phase of trail	Patients, n	Median age	Drug class intervention	Intervention and dosage	Treatment and dosage	Primary endpoint
1	Barlesi F et al. (2018) (14)	3	792	63.5	3	Avelumab 10mg/kg/2W	Docetaxel 75mg/m^2^/3W	PFS
2	Fehrenbacher L et al. (2016) (15)	2	287	62	1	Atezolizumab 1200mg/3W	Docetaxel 75mg/m^2^/3W	PFS
3	Garassino M C et al. (2013) (16)	2	219	66.5	2	Erlotinib 150mg/D	Docetaxel 75mg/m^2^/3W	PFS
4	Garon E B et al. (2014) (17)	3	1253	61.5	3	Ramucirumab 10mg/kg/3W + Docetaxel 75mg/m^2^/3W	Placebo + Docetaxel 75mg/m^2^/3W	PFS
5	Gerber D E et al. (2018) (18)	3	597	62.5	3	Bavituximab 3mg/kg/W + Docetaxel 75mg/m^2^/3W	Placebo + Docetaxel 75mg/m^2^/3W	PFS
6	Herbst R S et al. (2015) (19)	2&3	689	63	3	Pembrolizumab 10mg/kg/3W	Docetaxel 75mg/m^2^/3W	PFS
7	Jänne P A et al. (2017) (20)	2&3	510	61.4	2	Selumetinib 75mg/0.5D + Docetaxel 75mg/m^2^/3W	Placebo + Docetaxel 75mg/m^2^/3W	PFS
8	Kawaguchi T et al. (2014) (21)	3	301	68	2	Erlotinib 150 mg/D	Docetaxel 75mg/m^2^/3W	PFS
9	Kubota k et al. (2015) (22)	3	596	62	1	S-1 80mg/m^2^/D + cisplatin 60mg/m2/W	Docetaxel 60mg/m^2^/3W + Cisplatin 80mg/m^2^/3W	PFS
10	Lee D H et al. (2010) (23)	3	161	57.5	2	Gefitinib 250mg/D	Docetaxel 75mg/m^2^/3W	PFS
11	Manegold C et al. (2013) (24)	2	70	60.2	1	Cilengitide 600mg/m^2^/0.5D	Docetaxel 75mg/m^2^/3W	PFS
12	Ramlau R et al. (2012) (25)	3	913	59.6	2	(Ziv-) aflibercept 6mg/kg/3W + Docetaxel 75mg/m^2^/3W	Placebo + Docetaxel 75mg/m^2^/3w	PFS
13	Reck M et al. (2014) (26)	3	1314	60	2	Docetaxel 75mg/m2/3W + Nintedanib 200mg/0.5D	Docetaxel 75mg/m^2^/3W	PFS
14	Rittmeyer A t al. (2016) (27)	3	850	64	1	Atezolizumab 1200mg/3W	Docetaxel 75mg/m^2^/3W	PFS
15	Rodrigues-Pereira J et al. (2011) (28)	3	211	59.5	1	Pemetrexed 500mg/m^2^/3W + Carboplatin 5mg/ml/min	Docetaxel 75mg/m^2^/3W + Carboplatin 5mg/ml/min	PFS
16	Socinski M A et al. (2010) (29)	2	146	66	1	Pemetrexed 500mg/m^2^/3W + Carboplatin 6mg/ml/min	Docetaxel 75mg/m^2^/3W + Carboplatin 6mg/ml/min	PFS
17	Yoh K et al. (2016) (30)	2	157	65	3	Ramucirumab 10mg/kg/3W + Docetaxel 60mg/m2/3W	Placebo + Docetaxel 75mg/m^2^/3W	PFS
18	Pillai R N et al. (2019) (31)	3	672	68	2	Ganetespib 150mg/m^2^/2W + Docetaxel 75mg/m^2^/3W	Docetaxel 75mg/m^2^/3W	PFS

Drug class of intervention: 1- Antineoplastic agents, 2- Kinase inhibitors, 3- Monoclonal antibodies. W, week; D, day.

**Figure 2:**
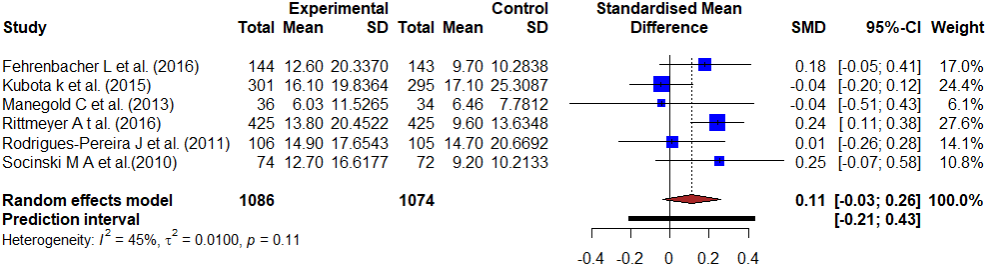
Forest plot representing the PFS of docetaxel versus antineoplastic agents treatment. Hedge’s corrected SMD is -0.36, and Higgin’s and Thompson’s I2 statistic is 96%.

**Figure 3:**
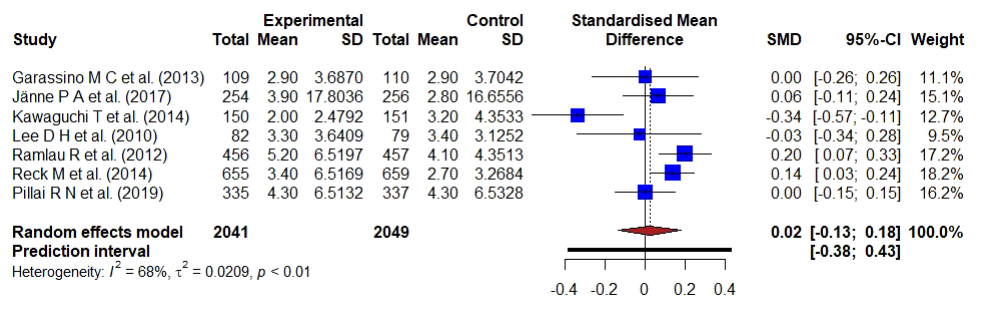
Forest plot representing the PFS of docetaxel- versus kinase inhibitors- treatment; the Hedge’s corrected SMD is 0.02, and Higgin’s and Thompson’s I2 statistic is 68%.

**Figure 4:**
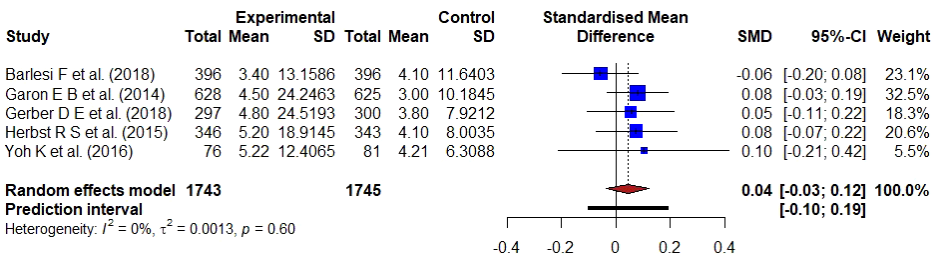
Forest plot representing the PFS of docetaxel versus monoclonal antibodies treatment. Hedge’s corrected SMD is 0.04, and Higgin’s and Thompson’s I2 statistic is 0%.

**Figures 2**-**4** show forest plots comparing the PFS of docetaxel to antineoplastic agents, kinase inhibitors, and monoclonal antibodies-based treatment. The six studies reported the PFS of antineoplastic agents compared with docetaxel with 2160 patients involved. The meta-analysis of all involved studies revealed significant statistical heterogeneity (I2 = 96%, τ2 = 0.2502, p < 0.01), and Hedge’s corrected SMD was -0.36 (95% CI: -1.01–0.29). There was a moderate effect because it was a negative value smaller than -0.20, which implies the result favored the antineoplastic agents-based treatment.

A total of 4090 patient data from seven studies reported the PFS of kinase inhibitor compared with docetaxel-based treatment. A bias-corrected SMD; Hedge’s g-value was 0.02 (95% CI: -0.13–0.18), implying the result favored the docetaxel-based standard treatment. A significant statistical heterogeneity (I2 = 68%, τ2 = 0.0209, p < 0.01) was found in the pooled analysis of all included studies.

The PFS of monoclonal antibodies was compared to docetaxel in five studies involving 3488 individuals. There was no substantial statistical heterogeneity in a pooled analysis of all included trials (I2 = 0, τ2 = 0.0013, p = 0.60), and Hedge’s g-value was 0.04 (95% CI: -0.03–0.12), indicating that the result favored docetaxel-based treatment. The SMD value was less than 0.20, indicating that docetaxel had a minor effect.

**Figure 5:**
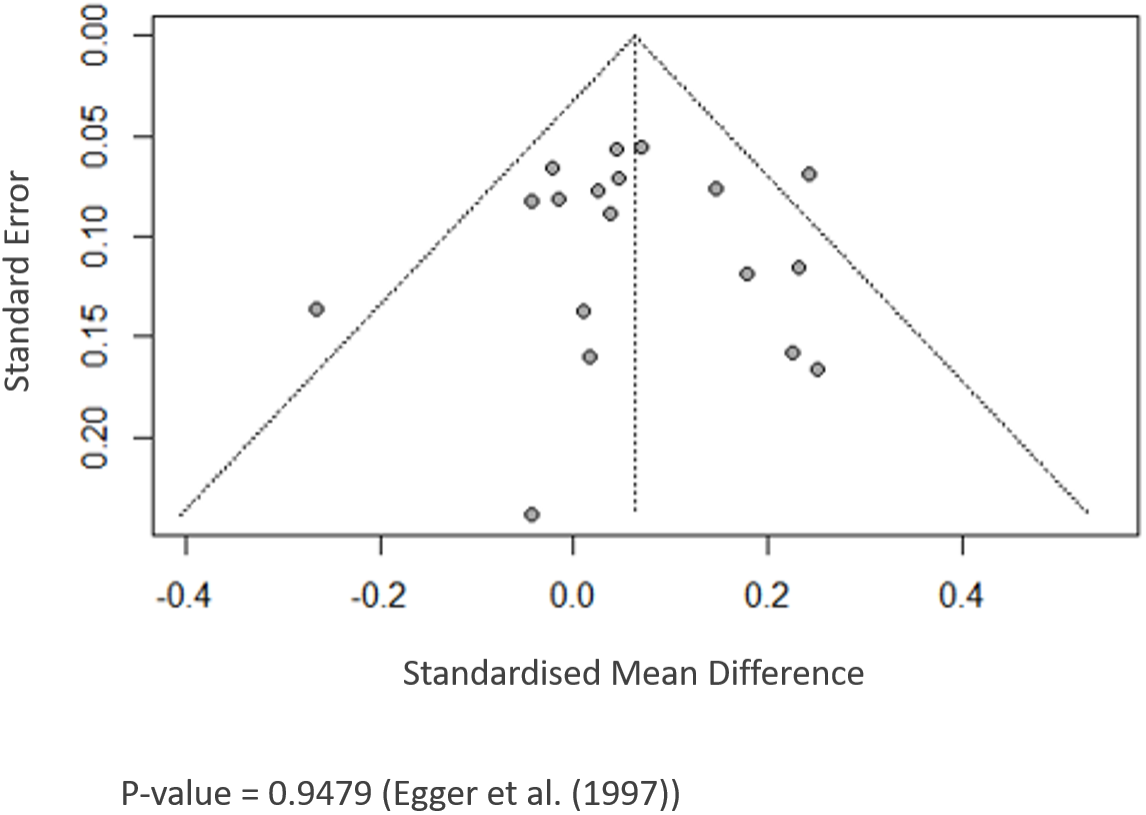
Funnel plot showing publication bias.

The p-values for the meta-analyses of PFS of 18 RCTs are > 0.05, indicating that formal statistical testing revealed no indication of significant publication bias (PFS: Egger’s test, p = 0.947) (**Figure 5**).

## 4 Discussion

A meta-analysis was conducted for 18 RCTs (14–31) with docetaxel as the experimental group and antineoplastic agent, and kinase inhibitor and monoclonal antibodies as a control group including 9738 patients with stage III–IV NSCLC. The objective of this study was to see if the PFS of patients improved. Platinum-based two-drug combinatorial chemotherapy has been the standard of care for advanced NSCLC patients (22–24). Our study’s main aim was to compare the two treatment regimens in terms of PFS in patients with advanced NSCLC (24). A total of 2160 cases with six RCTs were used to compare the docetaxel with antineoplastic agents. Six studies compared the improvement of PFS between docetaxel and atezolizumab, S-1 plus cisplatin, cilengitide, pemetrexed/carboplatin (15, 22, 24, 27–29). The period from randomization to either progressing illness or death was referred to as PFS. The different randomization methods are used to receive either 60 mg/m2 docetaxel plus cisplatin, 75 mg/m2 docetaxel, docetaxel 75 mg/m2/3W + carboplatin 5 mg/ml/min or oral S-1 80 mg/m2/day plus cisplatin 60 mg/m2, cilengitide 600 mg/m2, pemetrexed 500mg/m2/3W + carboplatin 5mg/ml/min, atezolizumab 1200 mg to see the improvement of PFS between these groups (15, 22, 24, 27–29). The PFS was similar between each control and treatment group. The median PFS was 2.7 months with atezolizumab and 3.0 months with docetaxel with a HR of 0.94 (95% CI 0.72–1.23) (15). The median PFS was 2.8 months with atezolizumab and 4.0 months with docetaxel. The HR was 0.63 [95% CI 0.43–0.91] (27). The median PFS was 4.9 months in the SP group and 5.2 months in the DP group with a HR of 1.113; 95% CI, 0.945 to 1.311 (22). There were no statistically significant differences in PFS between the treatment groups with a HR of 0.91 (0.67–1.23) (24). Therefore, there was no improvement in PFS between the groups.

In patients with metastatic NSCLC, antibodies targeting the immune checkpoint molecules PD-L1 or PD-1 enhance PFS compared to standard of care chemotherapy treatment (14). A total of 3488 patients in five trials have been used to compare docetaxel-based treatment with monoclonal antibody-based therapy (14, 17–19, 30). The meta-analysis of avelumab versus docetaxel in advanced NSCLC patients and progression of disease following platinum-based treatment was described by Barlesi et al. (14). A block randomized method was used to acquire either docetaxel 75 mg/m2 or avelumab 10 mg/kg and PFS was a secondary endpoint. The median PFS in the avelumab group was 2.8 months (95% CI 2.7–3.5) and 4.2 months (3.3–5.2) in the docetaxel group with HR 1.16 [95% CI 0.97–1.40]. As a result, with avelumab, PFS was substantially longer, and objective responses were more likely than with docetaxel. Garon et al., compared the effectiveness and safety of docetaxel with ramucirumab versus placebo as second-line therapy for stage IV NSCLC patients (17). A randomized method was used to assign the patients either ramucirumab 10 mg/kg or docetaxel 75 mg/m2. The median PFS for the ramucirumab group was 45 months, compared to 30 months for the control group with a HR of 0.76 (0.68–0.86). The PFS is improved in ramucirumab compared to docetaxel in patients with stage IV NSCLC. The efficacy of bavituximab in combination with docetaxel in patients with advanced NSCLC who have already been treated was investigated by Gerber et al. (18). The authors used a stratified randomized technique to provide docetaxel plus placebo or docetaxel plus bavituximab 3 mg/kg to the patients. With HR 1.00; 95% CI, 0.82–1.22, there was no alteration in PFS. The addition of bavituximab to docetaxel did not improve PFS. Herbst et al. compare pembrolizumab’s effectiveness and safety to those of docetaxel (19). A randomized method was used to acquire either pembrolizumab 10 mg/kg or docetaxel 60 mg/m to the selected participants. The median PFS was 3.9 months with pembrolizumab, 4.0 months with docetaxel (HR 0.88, 95% CI 0.74–1.05). Therefore, PFS was significantly longer with pembrolizumab than docetaxel. Yoh et al., explain how a phase II, double-blind, randomized, placebo-controlled trial in Japanese patients with NSCLC examined the safety and effectiveness of second-line ramucirumab-docetaxel (30). The median PFS was 5.22 months for ramucirumab-docetaxel and 4.21 months for placebo-docetaxel with HR of 0.83 (95% CI 0.59–1.16). Hence, PFS was longer with ramucirumab-docetaxel than with placebo-docetaxel. Seven clinical studies, including 4090 participants, were conducted to compare the docetaxel-based therapy with kinase inhibitor for patients with advanced NSCLC. The authors of seven studies compared the efficacy and safety of Gefitinib, erlotinib, aflibercept (Ziv-aflibercept), docetaxel plus nintedanib, mitogen-activated protein kinase (MEK) inhibitor, selumetinib + docetaxel and combination of ganetespib-docetaxel with the treatment group of docetaxel in patients with advance NSCLC to check the improvement of PFS between the groups. A randomized clinical method was used and patients received either docetaxel (75 mg/m2), IV placebo plus docetaxel (75 mg/m2), placebo + docetaxel (75 mg/m2/3W) or gefitinib (250 mg/d), erlotinib orally 150mg/day, (Ziv-) aflibercept 6 mg/kg intravenous plus docetaxel 75 mg/m2 erlotinib 150 mg/D, nintedanib 200 mg orally, selumetinib 75mg/0.5D plus docetaxel 75mg/m2/3W, ganetespib 150 mg/m until unacceptable side effects or disease progression based on previous bevacizumab treatment, histology, ECOG performance status, and presence of brain metastases (15, 19, 20, 22, 24, 25, 30). PFS was estimated as a primary and secondary endpoint in these studies. The median PFS was 3.9 months with selumetinib + docetaxel and 2.8 months with placebo + docetaxel with HR, 0.93 [95% CI, 0.77–1.12] (19). The median PFS in the ganetespib and docetaxel arm was 4.2 months, and 4.3 months in the docetaxel arm, with an HR of 1.16 (95% CI, 0.96–1.403) (31). Gefitinib had a better PFS than docetaxel, with a HR of 0.729; 90% CI, 0.533–0.998. The PFS was longer with gefitinib than docetaxel. As a result, gefitinib was a crucial and effective second-line treatment option for Korean NSCLC patients (23). Gefitinib had a longer PFS than docetaxel. The median PFS was 2.9 months with docetaxel versus 2.4 months with erlotinib with HR 0.71, 95% CI 0.53–0.95 (16). Median PFS was significantly longer in the (Ziv-)aflibercept arm of 5.2 months than in the placebo arm of 4.1 months with HR was 0.82 (95% CI 0.72–0.94). Erlotinib had a median PFS of 2.0 months against 3.2 months when compared to docetaxel with an HR of 1.22; 95% CI, 0.97 to 1.55. In an EGFR-unselected patient sample, erlotinib failed to improve PFS compared to docetaxel (21). The median PFS in the docetaxel plus nintedanib group was 3·4 months compared to 2.7 months in the docetaxel plus placebo group (HR 0.79, 95% CI 0.68–0.92) (26).

There are limits to our analysis that should be considered while evaluating the results. First, the different treatment regimens add to the meta-analysis’ clinical heterogeneity, which makes meta-analysis interpretation more difficult. In three studies, docetaxel was used in conjunction with other medicines, either cisplatin or carboplatin, in the control arm. The quality of the results was influenced by the quality of each study’s results. Finally, because the research included in this study was all conducted in the West, the findings must be confirmed in Asia. Docetaxel was revealed to be more effective in the second-line therapy of advanced NSCLC than antineoplastic drugs, kinase inhibitors, and monoclonal antibodies, according to the findings.

## 5 Conclusion

The phase 2 and 3 study of antineoplastic agents demonstrate a clinically significant survival benefit over docetaxel in patients with NSCLC. Compared to docetaxel, monoclonal antibodies and kinase inhibitors did not affect PFS in NSCLC patients. From the results of 18 trials involving 9738 patients, those who received docetaxel-based therapy had a significantly longer PFS than those who received kinase inhibitors or monoclonal antibodies. In the overall meta-analysis, patients in the standard treatment arm had a slightly longer PFS than those in the experimental therapy arm. Biological behavior subgroups such as those entirely refractory, those with partial and incomplete responses, and those with short and extended disease-free intervals will be examined in future meta-analysis investigations.

## Data availability statement

The original contributions presented in the study are included in the article/supplementary material. Further inquiries can be directed to the corresponding authors.

## Author contributions

CN: Methodology, Software, Writing – original draft, Writing – review & editing. AJ: Validation, Writing – original draft, Writing – review & editing. SC: Methodology, Validation, Writing – original draft. BS: Resources, Writing – review & editing. SR: Funding acquisition, Writing – review & editing. CD: Data curation, Writing – review & editing. KS: Validation, Writing – review & editing. SP: Resources, Supervision, Writing – review & editing. PS: Supervision, Writing – review & editing. PV: Supervision, Validation, Writing – review & editing. CSr: Data curation, Writing – review & editing. SK: Supervision, Validation, Writing – original draft. CSh: Conceptualization, Investigation, Supervision, Validation, Writing – review & editing.
